# Tunable resonant metasurfaces enabled by atomically thin semiconductors

**DOI:** 10.1038/s41377-026-02311-8

**Published:** 2026-07-10

**Authors:** Alexey Ustinov, Ángela Barreda, Duk-Yong Choi, Tobias Bucher, Giancarlo Soavi, Thomas Pertsch, Isabelle Staude

**Affiliations:** 1 Institute of Solid State Physics, Friedrich Schiller University Jena, Jena, Germany; 2 Institute of Applied Physics, Abbe Center of Photonics, Friedrich Schiller University Jena, Jena, Germany; 3 Department of Electronic Engineering, University Carlos III of Madrid, Leganés, Spain; 4 Laser Physics Centre, Australian National University, Canberra, ACT Australia; 5 Fraunhofer Institute for Applied Optics and Precision Engineering, Jena, Germany

**Keywords:** Other photonics, Nanophotonics and plasmonics

## Abstract

Nanophotonics has recently gained new momentum with the emergence of a novel class of nanophotonic systems consisting of resonant dielectric nanostructures integrated with single or few layers of transition metal dichalcogenides (2D-TMDs). Thinned to the single-layer phase, 2D-TMDs are unique solid-state systems with excitonic states able to persist at room temperature, demonstrating notable tunability in the optical frequency range. Based on these properties, 2D-TMDs offer important opportunities for hybrid nanophotonic systems where a tailored nanostructure serves to enhance the light-matter interaction in the 2D-TMDs, while the 2D-TMDs can provide various active functionalities, thereby dramatically enhancing the scope of these hybrid systems. In this work, we combine 2D-TMDs with resonant metasurfaces composed of high-index dielectric nanoresonators. The dependence of excitonic states in 2D-TMDs on the charge carrier density leads to an amplitude modulation of the corresponding optical transitions as the Fermi level varies, thereby altering the coupling strength between the 2D-TMD and the resonant modes of the photonic nanostructure. We experimentally implement such a hybrid nanophotonic system and demonstrate voltage tuning of its reflectance as well as polarization-dependent behavior. Our results show that hybridization with 2D-TMDs can serve to render resonant photonic nanostructures tunable - an important property for practical applications, e.g., in optical analog computers and neuromorphic circuits.

## Abstract (web summary)

Voltage-tunable hybrid metasurfaces integrating 2D transition metal dichalcogenides with photonic bound states in the continuum enable dynamic control of light-matter interaction and polarization-dependent optical responses for analog photonic applications.

## Graphical abstract

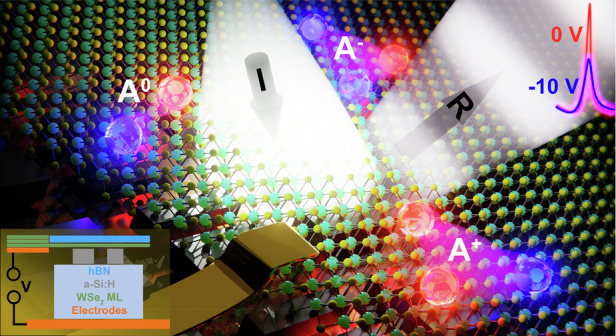

## Introduction

For decades, TMDs have been employed as a grease material in technical applications due to their low friction, as a result of the weak Van der Waals forces between their individual layers. However, when reduced to their monolayer form, TMDs exhibit a range of remarkable optical, mechanical, and electronic properties that position them as promising materials for future technologies^1,2^. Their intrinsic two-dimensional confinement and reduced dielectric screening result in a substantial exciton binding energy of up to 0.5 eV, allowing excitonic phenomena to be observed at room temperature^3–5^, while their direct bandgap enables intense photoluminescence (PL)^6^. Additionally, TMD monolayers demonstrate strong and tunable non-linear optical effects, allowing for the realization of advanced devices for all-optical modulation of polarization and wavefront shaping of light^7,8^.

The unique electronic structure of TMDs further enables the precise manipulation of their optical properties via external stimuli. In particular, their optical characteristics are highly tunable through chemical doping^9^, electrical gating^10–12^, optical fields^13,14^, mechanical strain^15^, changes in dielectric environment^16,17^, substrate effects^18^, and temperature variations^19,20^, among others. This adaptability underscores the potential of monolayer TMDs for the advancement of next-generation photonic, electronic, and optoelectronic systems^21,22^.

However, the single-atomic thickness of TMD monolayers limits the strength of light-matter interactions with incident optical radiation. This limitation can pose challenges for measuring their basic optical properties, such as absorption and reflection, and particularly their active response to external stimuli. Thus, for bare 2D-TMDs, the relative changes in these properties induced by such external stimuli are typically small and often become measurable only at cryogenic temperatures^23^. A way to overcome this challenge is to use hybrid systems, which rely on the coupling of 2D-TMDs to nanostructures able to confine optical fields in sub-wavelength volumes of the monolayer region^24–26^.

In this context, optical bound states in the continuum (BICs) supported by all-dielectric nanostructures present a particularly promising platform. BICs are unique modes that remain localized despite existing within the continuum spectrum of radiating states^27^. Initially introduced in quantum mechanics, their relevance has expanded to photonics, where they play a significant role in fields such as lasing, non-linear optics, sensing, chirality, and PL^28–32^. Theoretically, classical BICs are considered dark modes that neither couple to incident radiation nor emit it, exhibiting an infinite quality (*Q*)-factor^27^. High *Q*-factors are beneficial in achieving a strong light-matter interaction and, consequently, noticeable modulation amplitudes of optical properties within photonic structures supporting such states. However, these ideal non-radiative modes are purely mathematical constructs and cannot be observed directly in experiments. In practical implementations, the BIC transforms into a leaky mode, commonly referred to as a quasi-BIC (q-BIC) state^33^, showing high yet finite Q-factors.

The coupling of excitons in 2D-TMDs with q-BIC resonances in all-dielectric metasurfaces can significantly enhance their light-matter interaction. In this way, the utilization of the q-BIC state of a metasurface can be compared to previously demonstrated active hybrid structures based on Fabry-Pérot resonances for the enhancement of light-matter interaction in TMD monolayers^34^. The advantages of the metasurface-based approach include the possibility to manipulate the local optical field distribution, and thus the far-field radiation pattern by design, as well as the higher compactness, since light-matter interaction in such systems takes place at the nanoscale. Among demonstrated applications such as enhancement of PL emission^29^, second-harmonic generation^24,26,29^, low threshold^35^ and room-temperature lasing^36^, enhanced tuning effects are also expected in hybrid nanostructures. The control of optical properties in such hybrid structures relies on dynamically altering the effective refractive index of the TMD monolayer, which in turn induces changes in its spectral response. Specifically, the population of excitonic states in atomically thin TMDs is highly dependent on the Fermi level^23^, which can be manipulated by the application of an external stimulus, such as a constant electric field^37–39^. This modifies the Fermi level and the associated carrier density in the 2D-TMD, thereby altering the optical response of the semiconductor primarily through the following mechanisms: Coulomb screening, scattering effects, and charged exciton (trion) formation, while other related effects, such as Pauli blocking and bandgap renormalization, were reported to only weakly influence the exciton binding energy^11^. Notably, the additional charge carriers at elevated Fermi levels increase the Coulomb screening between electrons and holes, decreasing neutral exciton formation probability. This contributes to a shift of the optical response from neutral to charged excitons, effective spectral broadening, and a shift of the excitonic peak. As a consequence, both emission and absorption/reflection processes can be spectrally tuned by varying the external electric field strength. While other tuning approaches for all-dielectric metasurfaces, such as integration with phase change materials^40,41^ and liquid crystals^42^, as well as mechanical tuning^43^, have been demonstrated, exploiting 2D semiconductors as the active material paves the way for leveraging their other unique properties in future nanoscale devices with comprehensive active functionality. These include their strong direct-bandgap photoluminescence, excitonic response at room temperature, strong second-order nonlinear response, and rich options for integration and tailored responses via stacking^44^.

**Fig. 1:**
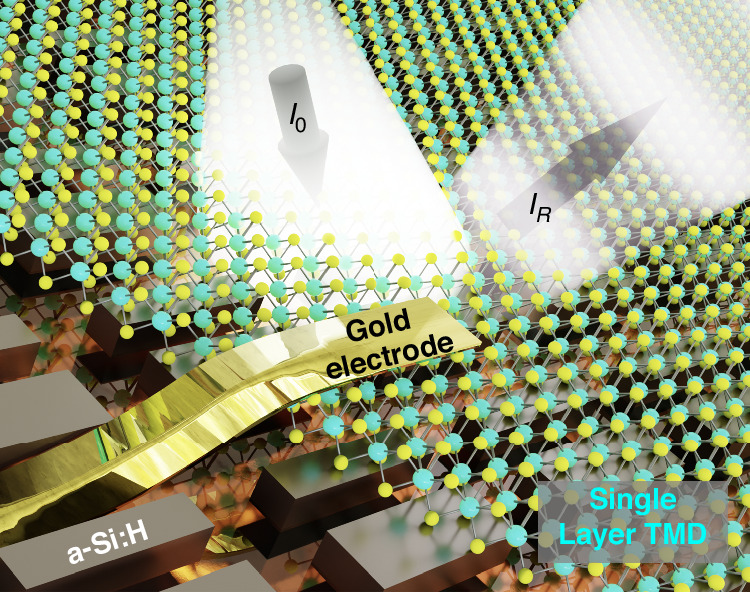
An artist’s impression of the hybrid resonant metasurface (not to scale). A WSe_2_ monolayer is positioned on top of a metasurface supporting a q-BIC state within the wavelength range of the excitonic resonance of the former. The top gold electrode allows for the injection of charge carriers and thus Fermi level control, leading to tunability of the linear-optical properties of the hybrid metasurface

In this work, we demonstrate numerically and experimentally the active tuning of a hybrid system consisting of a TMD (WSe_2_) monolayer coupled to a Mie-resonant metasurface. A conceptual sketch of the structure is shown in Fig. 1. Tunability is achieved through application of an external electric field, enabling dynamic control of the system’s linear-optical properties, specifically its absorptance and reflectance. By controlling the Fermi level in the WSe_2_ monolayer via an external electric field, we manipulate excitonic population distribution between neutral (A^0^) and charged (A^±^) states. This balance between excitonic states primarily governs the complex refractive index in TMD monolayers within the visible and near-infrared ranges of wavelengths at room temperature. The enhancement of the neutral A^0^-exciton increases the real part of the WSe_2_ monolayer’s optical conductivity, which subsequently leads to higher absorption. The coupling effect between the WSe_2_ monolayer and the dielectric metasurface, which exhibits a q-BIC resonance at the A^0^-exciton wavelength, leads to an enhancement of this modulation effect. As a consequence, we report a modulation depth of up to 20% in the reflection spectrum of the hybrid metasurface. The results of this work could serve as a platform for further investigation of exciton dynamics in monolayer TMDs and its interaction regimes with resonant photonic modes.

Moreover, our approach has the potential to enable ultrafast electrical tuning of the metasurface optical response, which is ultimately only limited by the electron relaxation time at the TMD monolayer/Au electrode interface (~1 ps)^45^ and can yield terahertz switching rates.

## Results

## Metasurface design and linear characterization

**Fig. 2:**
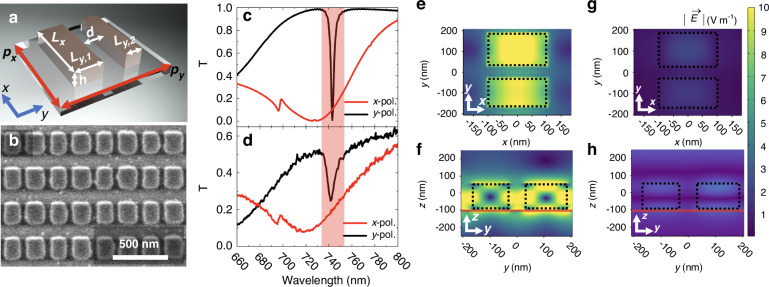
Q-BIC metasurface design and characterization. **a** Sketch of the q-BIC metasurface unit cell with denoted geometrical parameters. **b** SEM image of the fabricated q-BIC metasurface. **c** Simulated and **d** measured transmission spectra of the bare metasurface sample featuring a q-BIC-resonance at 742 nm for normally incident linearly polarized light. The red and black curves denote *x*- (off-resonant case) and *y*-polarized light (q-BIC excitation), respectively. The red shaded area outlines the excitonic band represented by neutral A^0^-exciton and charged A^±^-trions. **e**–**h** Near-field maps at *λ* = 742 nm for the polarization of the incident radiation (**e**, **f**) along the *y*- (q-BIC excitation) and (**g**, **h**) *x*-axis (off-resonant case) in (**e**, **g**) *XY*- and (**f**, **h**) *YZ*-planes. The *XY*-plane is positioned 1 nm above the nanobars, while the *YZ*-plane intersects the nanobars at their geometric center. The black contours represent the edges of the a-Si:H bars, and the red lines correspond to the substrate surface

The metasurface considered in this work is based on an established design for q-BIC supporting structures^26,46,47^ consisting of two asymmetric hydrogenated amorphous silicon (a-Si:H) nanobars per unit cell, located on a fused silica substrate. A sketch of the corresponding unit cell is shown in Fig. 2a, b, respectively. The different widths (*L*_*y*,1_ and *L*_*y*,2_) of the nanobars allow for the excitation of the q-BIC at the *Γ*-point in reciprocal space, originating from two in-plane magnetic dipoles oriented in opposite directions and resulting in a non-zero net magnetic dipole moment of the unit cell. The q-BIC-resonance wavelength is designed to coincide with the A^0^-exciton optical transition of the WSe_2_ monolayer at room temperature. For metasurface unit cell geometry optimization, we performed numerical simulations based on the finite-element method (FEM), and ellipsometrically measured data for the complex refractive index of a-Si:H. The refractive index of the substrate was taken as 1.45. The optimization resulted in a structure height *h* = 140 nm, meta-atom lengths along *x*- and *y*-directions *L*_*x*_ = 202 nm, *L*_*y*,1_ = 161 nm, *L*_*y*,2_ = 140 nm, separation between meta-atoms *d* = 57 nm, and periodicity along *x*- and *y*-directions *p*_*x*_ = 367 nm and *p*_*y*_ = 413 nm, respectively. The radiative Q-factor dependency on the asymmetry parameter of the metasurface unit cell is shown in Fig. S1, which evidences the presence of a q-BIC state excited at the designed wavelength. The near-field maps for the absence of asymmetry (*α* = 0) and small perturbations corresponding to *α* = 0.01 and 0.1 are represented in Fig. S2a–c, which evidence the excitation of q-BIC when the symmetry of the unit cell is broken. In fact, for asymmetric metasurfaces, we observe an out-of-plane electric dipole that comes from a pair of in-plane magnetic dipoles, oriented in opposite directions, resulting in a non-zero magnetic dipole moment of the unit cell (see Fig. S2b, c). As additional evidence of the symmetry-protected BIC nature of the resonant metasurface mode, transmittance spectra in Fig. S2d illustrate the absence of a radiative channel for a symmetric unit cell (*α* = 0) and appearance of the q-BIC state starting from a small asymmetry parameter value (*α* = 0.01).

The validation of fabricated metasurface parameters was performed by scanning-electron microscope (SEM) imaging (see Fig. 2c). Next, we conducted linear-optical transmission spectroscopy of the metasurface using a custom-built setup^48^. These results are shown in Fig. 2d and are in good agreement with our theoretical expectations (Fig. 2c, d). The sharp transmission dip observed for *y*-polarized incident light is associated with the excitation of the q-BIC state. For *x*-polarized incident light, a weaker resonant feature occurs around 700 nm wavelength, well below the spectral range of the excitonic resonance of WSe_2_ at room temperature centered around 742 nm (red shaded area in Fig. 2c, d). The near-field intensity profiles at *λ* = 742 nm are shown in Fig. 2e, g, f, h for *x*- and *y*-polarized incident light, respectively. For *y*-polarized incident light, the q-BIC resonance is excited, leading to an average electric-field intensity enhancement at 1 nm above the nanobar surface by a factor of 35 with respect to the incident field. In contrast, for *x*-polarized incident light, no q-BIC can be excited at 742 nm, resulting in a smaller average field intensity enhancement of 1.09 within the same plane. As such, we can expect the q-BIC resonance to enhance the interaction of light with a two-dimensional material situated on top of the metasurface.

## Hybrid metasurface assembly and static properties

**Fig. 3:**
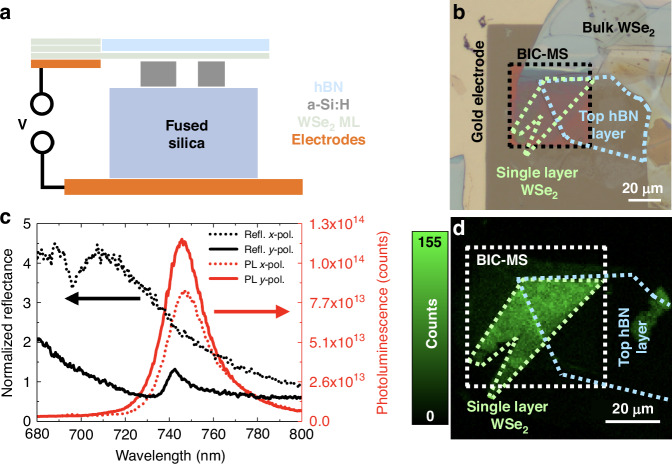
Hybrid metasurface design and characterization. **a** Schematic of the hybrid metasurface unit cell, including a top gold injection and a bottom copper ground electrode. The top electrode is in direct contact with the bulk part of the exfoliated TMD crystal, which in turn is connected to its monolayer part covered by a few-layer hBN. The potential difference between the electrodes leads to the shift of the Fermi level in the TMD monolayer (not to scale). **b** Top-view bright-field microscopy image of the hybrid resonant structure with the main constituents highlighted by dashed lines. The top hBN layer serves as WSe_2_ monolayer encapsulation. **c** Linear reflection (black) and PL emission (red) spectra of the hybrid resonant structure for *x*- (dots) and *y*- (solid curves) polarized detected light, respectively. The reflectance is referenced to the reflectance of the substrate. **d** PL emission map of the hybrid resonant structure. The outlines of the WSe_2_ monolayer, hBN layer, and the metasurface are marked for clarity

The schematic architecture of the hybrid metasurface is shown in Fig. 3a. The entire sample was placed onto a copper plate serving as a ground electrode. The WSe_2_ monolayer is in contact with the top gold electrode through the bulk part of the WSe_2_ material (depicted as a few layers of TMD material in Fig. 3a), and covered by a few-layer hexagonal boron nitride (hBN) crystal for protecting the monolayer above the active area of the metasurface structure. A bright-field microscopy image of the hybrid structure is represented in Fig. 3b with the main components denoted. The ground electrode positioned under the substrate (not visible in top view) prevents optical measurements in transmission geometry. An additional bright-field microscopy image showing a larger area surrounding the metasurface, including the upper electrode configuration, is presented in Fig. S3. Note that the influence of a few-layer hBN crystal on top of the structure used in our design can be considered negligible for its electromagnetic properties. For instance, calculations confirm that even relatively thick hBN layers (2-4 nm) would not noticeably modify the Q-factor of the q-BIC resonance of the metasurface. While they introduce a red shift in the transmission spectrum of only a few nanometers (Fig. S4), this shift is negligible when compared to the FWHM of the fabricated metasurface’s q-BIC state (≈10 nm) and that of the WSe_2_ monolayer’s excitonic resonance at room temperature (≈20 nm).

To optically characterize the hybrid system without any voltage applied, we measured its linear-optical reflection spectra for *x*- and *y*-polarized reflected light from the area of the monolayer on top of the metasurface. Corresponding results are displayed in Fig. 3c. The expected correspondence with the transmittance spectra in Fig. 2c is clearly observed, with peaks transforming into dips and vice versa. Note that the reflectance spectra are referenced to the signal from the fused silica substrate, including the ground electrode (Fig. S5), in order to correct for the influence of the substrate on the measurements. Moreover, we collected polarization-resolved room-temperature PL spectra from the same region. These results are also shown in Fig. 3c. The small spectral blue shift observed for the PL peak upon changing the linear polarization of detection from *x*-polarized (748 nm) to *y*-polarized (745 nm) could be the first indication of the interaction between the monolayer WSe_2_ and the metasurface. The observed blue shift could also be induced by possible mechanical strain in the system due to a nanostructured geometry of the metasurface. When compared to heterostructures fully supported by a uniform dielectric substrate, additional charge doping, changes in dielectric screening, Purcell enhancement, and directionality effect^49^ could contribute to the exact PL spectrum of the hybrid metasurface. At room temperature, the coupling between the metasurface modes and spectrally broadened PL transitions set within the TMD monolayer is reduced. Consequently, any difference in the PL enhancement factors between the two polarization states is expected to remain within an order of magnitude, which is in line with our experimental observations. A corresponding PL map of the system is shown in Fig. 3d. Outlined are the components of the solid-state part of the hybrid system, i.e., the WSe_2_ monolayer and auxiliary insulating layer of hBN used to separate the monolayer from the atmospheric influence and inhibit its chemical degradation and uncontrolled charge doping. The bright area in the PL map corresponds to the spatial overlap between the metasurface and the monolayer and is caused by the directivity properties of the metasurface, which facilitates efficient collection of the PL signal.

## Electrical gating

Next, we investigated the behavior of the hybrid system for a variation of the Fermi level of the monolayer subsystem. All experiments were conducted at room temperature.

**Fig. 4:**
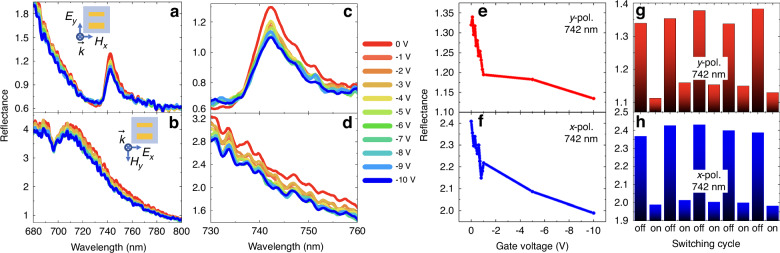
Linear reflectance evolution under external voltage. **a**, **b** Linear reflectance spectra for two polarization states of excitation as depicted in the insets. Scaled-up reflectance spectra near the A^0^-excitonic transition at 742 nm demonstrating the change of reflectivity for (**c**) the q-BIC and (**d**) off-resonant mode, when the external voltage changes from 0 V to −10 V. The reflectance values at 742 nm for different values of the external voltage and for (**e**) q-BIC mode excitation and (**f**) off-resonant cases. **g**, **h** The reflectance values at 742 nm for a sequence of external voltage switching cycles when the A^0^-exciton oscillator strength is suppressed (off-state) and restored (on-state). All reflectance spectra are referenced to the reflectance of the bare substrate measured under the same conditions

To this end, we measured its linear-optical reflection spectra using the same setup as described above, but with an external voltage applied between the top injection and bottom ground electrode by an external voltage source (Rohde & Schwarz NGA 141). Thereby, a negative applied voltage refers to the negative ("-") output of the voltage supply connected to the bottom contact and the positive ("+") output connected to the top injection electrode. These results are shown in Fig. 4a, b for *y*- and *x*-polarized reflectance, respectively, and for different applied voltages.

The external voltage restoring the neutral A^0^-exciton absorption characteristics was found to be -10 V. While WSe_2_ monolayers are usually naturally p-doped^50^ and thus would require a positive external voltage to reduce the population of the positively charged trions, this polarity can be explained by initial n-doping from the substrate and surrounding environment. As such, the “switch-on" state of the neutral exciton is attained for this value, and the hybrid structure reaches a global reflectance minimum in the q-BIC resonance. At the lower Fermi level of the WSe_2_ monolayer (V_g_ = −10 V), i.e., when the optical properties of the latter are primarily governed by neutral exciton transitions, the absorption rate on the corresponding transitions increases, leading to a reflection reduction observed near 742 nm (Fig. 4a, b). In other words, the oscillator strength enhancement of the neutral A^0^-exciton results in an increase in the real part of the 2D conductivity of the WSe_2_ monolayer, which, in turn, leads to an increase in absorption.

We observe that both *x*- and *y*-polarized reflectance signals can demonstrate the modulation depth of up to ≈20% (Fig. 4a, b). The scaled-up regions of the reflectance spectra centered around the A^0^-exciton transition for two different excitation schemes (linear polarization of the incident radiation along the *y*- and *x*-axis), and a range of external voltage values ranging from 0 V to −10 V, with a step of 1 V are represented in Fig. 4c, d. As can be observed from the switching dependencies, the evolution of reflectance with external voltage is strongly influenced by the resonant coupling between the photonic mode of the metasurface and the excitonic transitions. The reflectance value evolution at the A^0^-exciton transition (742 nm) as a function of the external voltage steps is provided in Fig. 4e, f. The reflectance modulation results in Fig. 4a–f represent average dependencies of 5 subsequent experimental series under the same conditions.

The broad reduction in reflectance across the spectrum, starting from 680 nm, becomes particularly pronounced in the case of off-resonant excitation (Fig. 4b) and can be attributed to the presence of additional Mie resonances in the case of *x*-polarized excitation of the metasurface (see Fig. S6 for a multipole decomposition).

In order to test the repeatability of the switching mechanism, we performed measurements over five subsequent switching cycles. The corresponding results for the reflectance value at 742 nm wavelength are shown in Fig. 4g, h for *y*- and *x*-polarized reflected light, respectively. The depth of reflectance value modulation is comparable at a fixed wavelength of 742 nm for both types of excitation, but the switching process is more pronounced for the case of the q-BIC resonance (*y*-polarization) within the excitonic band compared to the off-resonant case (*x*-polarization) (Fig. 4c, d). This highlights the importance of coupling between the electronic and photonic subsystems for achieving an enhanced reflectance modulation depth while manipulating the electronic system state in the atomically thin material. In order to further increase the contrast of the switching, one could utilize a higher-Q-factor metasurface for achieving enhanced coupling strength between the two subsystems. Moreover, for real-world applications of such active photonic structures, it would be of critical importance to enhance the stability of the switching process, which may be achieved by optimization of the electronic periphery of the hybrid structure.

## Numerical modeling of the hybrid metasurface

In this section, we discuss the principles underlying the operation of the hybrid metasurface and the impact of charge doping on the 2D-conductivity of the TMD monolayer, which is accompanied by changes in the reflectivity and absorbance properties of the hybrid structure. Our numerical model accounts for the influence of the single-atomic-layer of WSe_2_ positioned on top of the q-BIC metasurface, which was initially optimized to match the q-BIC resonance with the A^0^-excitonic resonance of the WSe_2_ monolayer (see section Metasurface Design and Linear Characterization). To this end, we employ a semi-empirical model of 2D conductivity in the monolayer TMD^51^:

1 $${\sigma }_{{\rm{2D}},n}(\omega )=-i{\varepsilon }_{0}\omega {h}_{{\rm{eff}}}\cdot ({\varepsilon }_{{\rm{r}},n}(\omega )-1)$$

where *n* ∈ {*xx*, *xy*, *yx*, *yy*} denotes matrix components within the *XY*-plane of the TMD monolayer, *ε*_0_ is the dielectric permittivity of the classical vacuum, *ω* is the angular frequency, *h*_eff_ ≈ 6.49 Å corresponds to the effective WSe_2_ monolayer thickness^52^, and ${\widehat{\varepsilon }}_{{\rm{r}},n}(\omega )$ is a component of its dispersive complex dielectric permittivity tensor.

As the TMD monolayer virtually represents a 2D system while being three atoms thick, according to the unit cell geometry of the ${D}_{3h}^{\sigma }$ point group, the properties of such crystals can be described by effective surface quantities, such as surface conductivity^52^ or second-order nonlinear sheet susceptibility^10^. Through normalization to an effective thickness of a single monolayer, these quantities correspond to conventional bulk material properties, such as dielectric permittivity^52^ and second-order optical susceptibility^10^, accordingly. Here, we assume the absence of magneto-optical effects, so that the conductivity tensor of the TMD monolayer is diagonal. Given the 2D nature of the material, the out-of-plane components vanish:

2 $${\sigma }_{{\rm{2D}}}=\left[\begin{array}{cc}{\sigma }_{xx} & 0\\ 0 & {\sigma }_{yy}\end{array}\right]$$

where *σ*_*xx*_ = *σ*_*yy*_ since the linear optical response of the WSe_2_ monolayer is isotropic under experimental conditions^52^. The model includes inter-band contributions of the electronic system to the optical properties of the monolayer, which effectively describes semiconductor behavior of the latter and takes into account the excitonic contribution to dielectric permittivity in the form of the Lorentz oscillator model.

Each Lorentz oscillator in the model describes excitonic absorption/reflection peaks that would be observed experimentally. As mentioned above, when external voltage is applied, the Fermi level of the TMD monolayer shifts according to the polarity of the resulting potential difference between the TMD monolayer and the ground electrode. In the literature^53,54^, a conversion of the applied voltage to Fermi energy is often performed based on a charge injection or capacitance model, the latter yielding the simple relation E_F_ = *ℏ*^2^*π*CV_g_/2m*e with C being the back-gate capacitance, V_g_ – the applied voltage, m* ≈ 0.36 *m*_e_ – the effective electron mass^4^, and *e* – the elementary charge. However, for more complex experimental settings, as in our case, this simplified model might not be suitable. Instead, a quantitative description will require substantial new developments on the theoretical side. The frequency-dependent permittivity of the monolayer material described by the Lorentz oscillator model is given by^53^:

3 $${\varepsilon }_{{\rm{r}}}(\omega )={\varepsilon }_{\infty }+\mathop{\sum }\limits_{j}\frac{{a}_{j}{\omega }_{{\rm{P}}}^{2}}{{\omega }_{j}^{2}-{\omega }^{2}-i\omega {\gamma }_{j}}$$

where *a*_*j*_, *γ*_*j*_, and *ω*_*j*_ are the *j*th oscillator strength, damping factor, and resonant frequency, respectively, *ε*_*∞*_ = 1 corresponds to the static permittivity and *ω*_P_ = 28.3 meV is the plasma frequency, which depends on the carrier concentration. However, it does not change significantly within the range of electric field values used in this work.

With the known oscillator strength and static material parameters, the permittivity of a 2D material can be transformed into its optical conductivity using Eq. (1). This transformation enables the material to be treated as a purely 2D sheet while incorporating its optical properties. This approach provides a powerful formalism for modeling excitonic dynamics in external electromagnetic fields and facilitates the optical simulations of hybrid photonic structures^55^.

The parameters *a*_*j*_, *γ*_*j*_, and *ω*_*j*_ can be obtained either from first-principle calculations or experimentally by performing regression optimization of the model parameters to fit measured absorption, reflection, or transmission spectra. In the current work, we resort to the known tunability properties of a WSe_2_ monolayer with applied external voltage^37,52^ and implement an ad hoc excitonic tunability model. The free parameters of the model in the initial switching state were obtained by fitting to the experimental reflectance spectrum of the hBN-WSe_2_ monolayer heterostructure on fused silica at V_g_ = 0 V. Under current experimental conditions, the voltage-dependence of the oscillator strength of A^0^-state defines the tunable optical properties of the WSe_2_ monolayer and is represented in the model by a fully suppressed (*a*_1_ = 0) and restored (*a*_1_ = 1.858) A^0^-state. The contribution of oscillators corresponding to trions and higher-energy states (B- and C-excitons) to the reflectance amplitude modulation effect was a few orders of magnitude lower than that of the neutral exciton and was not observed in the experiment. The corresponding oscillator parameters are fixed in the Lorentz oscillator model and represent a background contribution to the optical properties of the WSe_2_ monolayer.

The values of the Lorentz oscillator parameters are summarized in Table 1. These parameters include oscillators corresponding to neutral exciton A^0^ (*j* = 1) for V_g_ = 0 V (fully suppressed A^0^-state) and − 10 V (restored A^0^-state) and trions A^±^ (*j* = 2), as well as the higher-energy *B*- (*j* = 3) and *C*-states (*j* = 4).

**Table 1:** Parameter values of the Lorentz oscillator model for WSe_2_ monolayer at room temperature for external voltage V_g_ varying from 0 V to -10 V

Oscillator number	*a*_*j*_	*ω*_*j*_ (eV)	*γ*_*j*_ (eV)
*j* = 1	0.0−1.858 × 10^3^	1.67	0.046
*j* = 2	6.26 × 10^2^	1.653	0.068
*j* = 3	6.437 × 10^3^	1.919	0.098
*j* = 4	1.439 × 10^5^	2.485	0.313

**Fig. 5:**
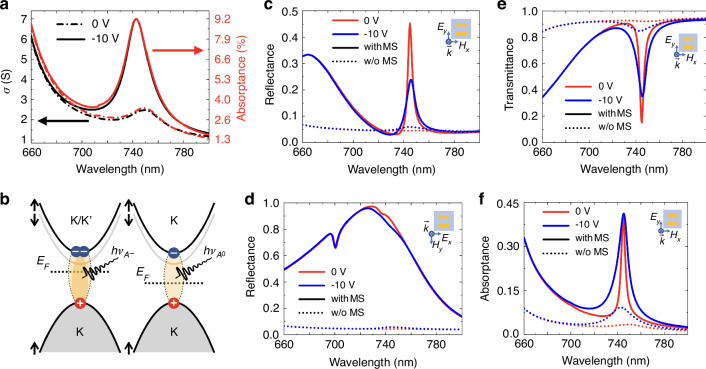
Calculated optical properties of a WSe_2_ monolayer and the hybrid metasurface. **a** Two-dimensional conductivity values, calculated based on the 2D-conductance model for two applied voltage values (black), along with the corresponding absorptance spectra of the WSe_2_ monolayer without q-BIC metasurface (red). **b** Illustration of the Fermi level control mechanism of the A^0^-exciton and A^±^-trion balance in the optical response of a WSe_2_ monolayer. It demonstrates the restoration of the A^0^-exciton upon the Fermi level shift to the middle of the bandgap. Calculated (**c**, **d**) reflectance, (**e**) transmittance, and (**f**) absorptance spectra of the heterostructure with (solid lines) and without q-BIC metasurface (dotted lines) upon two different voltage values for (**c**, **e**, **f**) the q-BIC and (**d**) off-resonant cases. The excitation polarization schemes are illustrated in the insets

The real part of the sheet conductivity of the optimized numerical model is shown in Fig. 5a. Taking into account the initial doping level of the WSe_2_ monolayer, switching the external voltage from 0 V to −10 V restores the A^0^-exciton contribution to the 2D conductivity. This restoration leads to an effective blue shift of the overall excitonic peak and an increase in its amplitude, resulting in higher absorption, as shown in Fig. 5a. The schematic band structure and two different states of the electronic system of the WSe_2_ monolayer (A^0^-exciton and A^−^-trion) are represented in Fig. 5b. When the Fermi level is shifted from the position near the lower conduction band (strong A^−^-trion) to the middle of the band gap, it corresponds to a restored population of A^0^-excitons, as the charge densities of electrons and holes become equalized. When the negative external voltage is further increased, and the Fermi level shifts towards the upper valence band, the relative concentration of positively charged holes effectively grows, leading to a higher probability of forming the positively charged trionic state A^+^. The bright A^−^-trions are formed by one hole in the $K({K}^{{\prime} })$-valley and two electrons from the same $K({K}^{{\prime} })$ or different ${K}^{{\prime} }(K)$ valleys with the exception of the inter-valley state with electrons occupying lower spin sub-bands^37^. The presence of such complex quasi-particles results in a lower spectral contribution, i.e., a lower oscillator strength, of the A^0^-state, which, in its turn, entails its reduced radiative decay rate. As a result, trionic states in monolayer TMDs manifest in their optical properties as broader and weaker resonances compared to neutral excitons^54^. A higher negative applied voltage facilitates the development of positively charged trions A^+^, which have a binding energy nearly identical to that of A^−^-state (within ~1 meV difference). As a result, A^+^-trions exhibit effectively the same total energy and contribute similarly to the 2D dielectric function of the WSe_2_ monolayer. This implies that, when compensated for the initial shift of the Fermi level in the WSe_2_ monolayer crystal, the optical properties of the hybrid metasurface would exhibit similar behavior for low doping levels in both negative and positive external voltage ranges at room temperature. The model was adjusted in accordance with the experimental observations of the modulation behavior of the A^0^-state in order to represent the specific heterolayer under investigation and its initial charge doping level, which may arise due to fabrication conditions and surrounding medium influence.

The increase in oscillator strength of the neutral A^0^-exciton leads to higher absorption in the WSe_2_ monolayer and, as a result, to a lower reflection coefficient of the hybrid metasurface within the spectral range of the excitonic transition (Fig. 5c, d). Although the effect is observed for both the q-BIC mode and off-resonant cases, the influence on the q-BIC mode is particularly pronounced reaching ≈50% for excitonic transitions coupled to the q-BIC state. An even stronger modulation effect could be achieved with a higher *Q*-factor resonance and at lower temperatures, which would reduce the thermal broadening of excitonic peaks.

Our numerical model demonstrates good qualitative agreement with experimental results for the hybrid metasurface, as evident from measured and calculated reflectance spectra shown in Figs. 4a, b, 5c, d, correspondingly. Thus, it empirically describes the excitonic tuning of a WSe_2_ monolayer and its influence on the reflectance change of the hybrid metasurface upon application of external voltage.

Predicted with our simulations, the modulation depth of the reflectance for the case of the excited q-BIC state reaches ≈50% within the excitonic bandwidth, while for the case when there is no resonant mode excited at the A^0^-excitonic transition, it reaches ≈5%. This suggests that the reflectance modulation depth is enhanced by the q-BIC resonance at the A^0^-exciton spectral range. In experiments, we observe that both cases can demonstrate the modulation depth of up to ≈20% (Fig. 4a, b), which can be explained by fabrication imperfections of the metasurface sample, the presence of the bottom reflective contact, and the influence of higher-energy quasi-particle states in the WSe_2_ monolayer crystal^53^ that were not considered in the numerical model.

Additional calculation results for transmittance and absorptance spectra behavior under the same external voltage values are represented in Fig. 5e, f, for the heterostructure with and without q-BIC metasurface. Both transmittance and absorptance values experience enhancement as a result of the influence of the q-BIC mode compared to the case of the metasurface absence. As can be observed by comparing calculated modulation of reflectance, transmittance, and absorptance spectra of the hBN-WSe_2_ heterostructure and of the hybrid metasurface (Fig. 5c, e, f), the modulation depth for the former reaches only ≈1% for external voltage varying in the range from 0 to -10 V and does not show a significant effect in room temperature measurements (Fig. S7). This is in contrast to the hybrid metasurface, which demonstrates an increase in modulation depth of reflectance at the excitonic transition by a factor of ≈15 (Fig. 4a, c). Although the absorptance amplitude of the hybrid metasurface does not undergo noticeable modulation, altered coupling conditions between the q-BIC mode and the A^0^-exciton lead to the modulation of transmission/reflection ratio at the q-BIC resonance within the excitonic spectral range. For instance, the simulation results for reflectance (Fig. 5c) clearly show the important role of resonance Q-factor (varying from 170 to 93) on tuning reflectance amplitude (changing from 45% to 24%).

## Discussion

We have experimentally demonstrated voltage tuning of the resonant metasurface enabled by the TMD monolayer. To this end, we hybridized a dielectric metasurface supporting the q-BIC resonance with the monolayer crystal of WSe_2_ featuring a neutral A^0^-excitonic state. Application of external voltage allowed us to reversibly change the reflectance of the hybrid metasurface. The effect is enhanced by the coupling between the WSe_2_ and the q-BIC resonances. While the effect remains small, reaching only ≈20% modulation of the initial reflectance level at the q-BIC resonance, it may be enhanced in the future by improved designs and integration strategies, further strengthening the coupling between the photonic mode and the excitonic resonance, i.e., by increasing the Q-factor of the q-BIC resonance (compare with Fig. S8). Our experimental results are underpinned by numerical simulations, which empirically describe the excitonic dynamics in the WSe_2_ monolayer and its influence on the linear reflectance change of the hybrid metasurface with an applied voltage. A good overall agreement is obtained between experimental and numerical results, positioning our model as a useful tool for describing and prototyping tunable resonant photonic structures based on Fermi level changes in 2D-TMDs.

The discrepancy in terms of relative modulation depth between calculation and experiment can be partly explained by the modest Q-factor achieved with the fabricated metasurface sample. It should be noted that the q-BIC has the potential to further enhance the effect by increasing the Q-factor. For comparison, Fig. 5c, d includes the calculated reflectance for the monolayer without a metasurface. The observed reflectance change between 0 V and −10 V of applied voltage is close to one percent only and below the noise level for our experimental conditions (Fig. S7), which additionally underscores the importance of the resonant photonic modes for the effect enhancement.

Moreover, a more in-depth study based on first-principles calculations, such as those based on *GW*-BSE method^56,57^, would be beneficial for the fundamental understanding of the coupling process between excitonic states in monolayer TMDs and resonant photonic modes in such hybrid photonic structures as presented in this work.

Altogether, our work introduces a new class of actively tunable photonic nanostructures that may find practical applications as adaptive optical elements, e.g., in analog computing systems or optical neural networks. For example, the proposed design can be employed as an amplitude modulation element for defining programmable weights in diffractive deep neural networks^58^ and reconfigurable memory in analog optical computing^59^. In addition, the possibility of manipulating the absorption and reflection of light with a few-100 nm layer in the visible spectral range can be beneficial for AR/VR systems^60^.

## Methods

## Numerical simulations

All numerical simulations in this work were performed using the commercial software package COMSOL Multiphysics 6.0 (Wave Optics module), which is based on the FEM Maxwell equations solver^61^. The optical response of the WSe_2_ monolayer was included as a conductive 2D-surface atop the metasurface. The 2D sheet conductivity model is based on Eqs. (1) and (2).

## Linear optical characterization

For measuring linear-optical reflection spectra, we used a stabilized free-space light source (Thorlabs SLS303) integrated with a reflection-geometry microscope based on ZEISS Axio Observer. The sample orientation was controlled by moving the stage of the microscope. To spatially filter the light reflected from the sample, an adjustable rectangular-shaped iris was placed in the focal plane of the objective, which collected the reflected signal from the sample. The quality of the filtered signal was controlled by a CCD-camera. After filtering, the signal was coupled into a multimode fiber connected to a spectrometer (ANDOR Kymera 328i-D2-sil). The measured spectra were normalized to the reflection from a clean region of the bare substrate (fused silica), including the bottom electrode.

The active time of each state corresponding to a particular voltage value was fixed at 5 seconds during the measurement. The limitation is based on hardware switching time and was chosen for the stability of potential values between the electrodes of our device. The fundamental switching time is limited by the Schottky barrier at the contact region between the top gold electrode and the TMD monolayer^45^. The total time of each measurement, including several (6–8) forward and backward switching cycles, averaged approximately 12 hours. The sample demonstrated reproducible results over 10 measurements within the first 6 months after fabrication. Further measurements demonstrated a noticeable decrease in the reflectance switching functionality of the hybrid structure due to the oxidation of the WSe_2_ monolayer. The durability of systems based on a similar design can be improved by the substitution of the WSe_2_ with more inert TMD materials and complete encapsulation of a monolayer crystal.

PL spectra were acquired using a commercially available PicoQuant MicroTime 200 confocal microscopy platform in combination with the same spectrometer.

## Nanofabrication

The fabrication of the silicon-based q-BIC structure started with the deposition of a 135-nm-thick a-Si:H film on a silica substrate. To this end, plasma-enhanced chemical vapor deposition (PECVD) was carried out in a Plasmalab 100 system from Oxford Instruments in which silane (SiH_4_) precursor and helium (He) dilution gas were used. To define the desired features of the metasurface, a positive electron beam resist (ZEP520A from Zeon Chemicals) was spin-coated onto the sample. In order to prevent electron charging during the electron-beam exposure process, a coating of Espacer (300Z from Showa Denko) was applied on the resist. The nano-bar patterns were written using a Raith150 system, followed by the development of the resist in ZED-N50. Then, a 30-nm-thick aluminum film was deposited on the sample through electron-beam evaporation (Temescal BJD-2000), and was patterned by removing the resist with resist remover (ZDMAC from Zeon Co.). The obtained aluminum patterns served as a hard etch mask during plasma etching of a-Si:H. The etch process was carried out using a fluorine-based inductively coupled plasma reactive ion etching system (Oxford Plasmalab System 100), where the etching conditions were optimized by precisely controlling the mixing ratio of CHF_3_ and SF_6_ gases for a vertical etch profile. Subsequently, aluminum wet etchant was applied to remove any residual aluminum from the patterned a-Si:H structure. The sample under investigation does not include an index-matching superstrate layer, since its presence would hinder the coupling between the metasurface modes and the TMD monolayer deposited on top of the structure.

The electrodes for WSe_2_ monolayer doping level control were fabricated by means of a DMD-based maskless lithography system (Microlight3D Smart Print UV) that was used for printing a positive mask (AZ1518 positive photoresist) for physical vapor deposition (PVD) of a 30-nm-thick gold layer. The printing resolution was 2 *μ*m. A lift-off process of the remaining metal layer was conducted in acetone and assisted by ultrasonication over several hours.

Following the procedure similar to ref. ^62^, hybridization of the metasurface with the 2D material was performed using the tape exfoliation method on WSe_2_ and hBN bulk crystals (HQ Graphene). The thickness of the exfoliated layers was controlled by contrast measurements^63^ using an optical microscope in the visible range. The monolayer quality was double-checked with PL measurements. The exfoliated crystals were collected layer by layer with a combined polydimethylsiloxane (PDMS)-polycarbonate (PC) stamp, ensuring clean and robust adhesion for pick-up and deposition of collected multi-layered systems onto various substrate materials and nanostructures. The deposition of the multi-layered structure onto the metasurface was carried out at a constant substrate temperature of 150 ^∘^C and kept at this elevated value for several hours to allow the PDMS-PC stamp to stabilize and the PC layer to detach from the PDMS part. After deposition with the PC film, the multi-layered structure was cleaned in trichloromethane (chloroform) solution for several hours until the PC layer was completely dissolved.

## Supplementary information

Supplementary Information

## Data Availability

The data is available from the authors upon reasonable request.
