# Supplementary material for: Tunable resonant metasurfaces enabled by atomically thin semiconductors
Source: Light Sci Appl. 2026 Jul 10;15:311. doi: 10.1038/s41377-026-02311-8 (PMC13354806; doi:10.1038/s41377-026-02311-8)
Supplement: Supplementary file 1 — Supplementary Information [file 41377_2026_2311_MOESM1_ESM.pdf]

Supplementary information: Tunable Resonant Metasurfaces Enabled by Atomically Thin Semiconductors

Alexey Ustinov,[*,†,‡] Ángela Barreda,[¶] Duk-Yong Choi,[§] Tobias Bucher,[†,‡] Giancarlo Soavi,[†] Thomas Pertsch,[‡,‖] and Isabelle Staude[†,‡]

†*Institute of Solid State Physics, Friedrich Schiller University Jena, Max-Wien-Platz 1, 07743 Jena, Germany*

‡*Institute of Applied Physics, Abbe Center of Photonics, Friedrich Schiller University Jena, Albert-Einstein-Str. 15, 07745 Jena, Germany*

¶*Department of Electronic Engineering, University Carlos III of Madrid, Avda. de la Universidad, 30, 28911 Leganés, Spain*

§*Laser Physics Centre, Australian National University, Canberra, ACT 2601, Australia*

‖*Fraunhofer Institute for Applied Optics and Precision Engineering, Albert-Einstein-Str. 7, 07745 Jena, Germany*

E-mail: alexey.ustinov@uni-jena.de

References

1. Evlyukhin, A. B. and Chichkov, B. N. Multipole decompositions for directional light scattering. *Physical Review B* **100**, 125415 (2019).

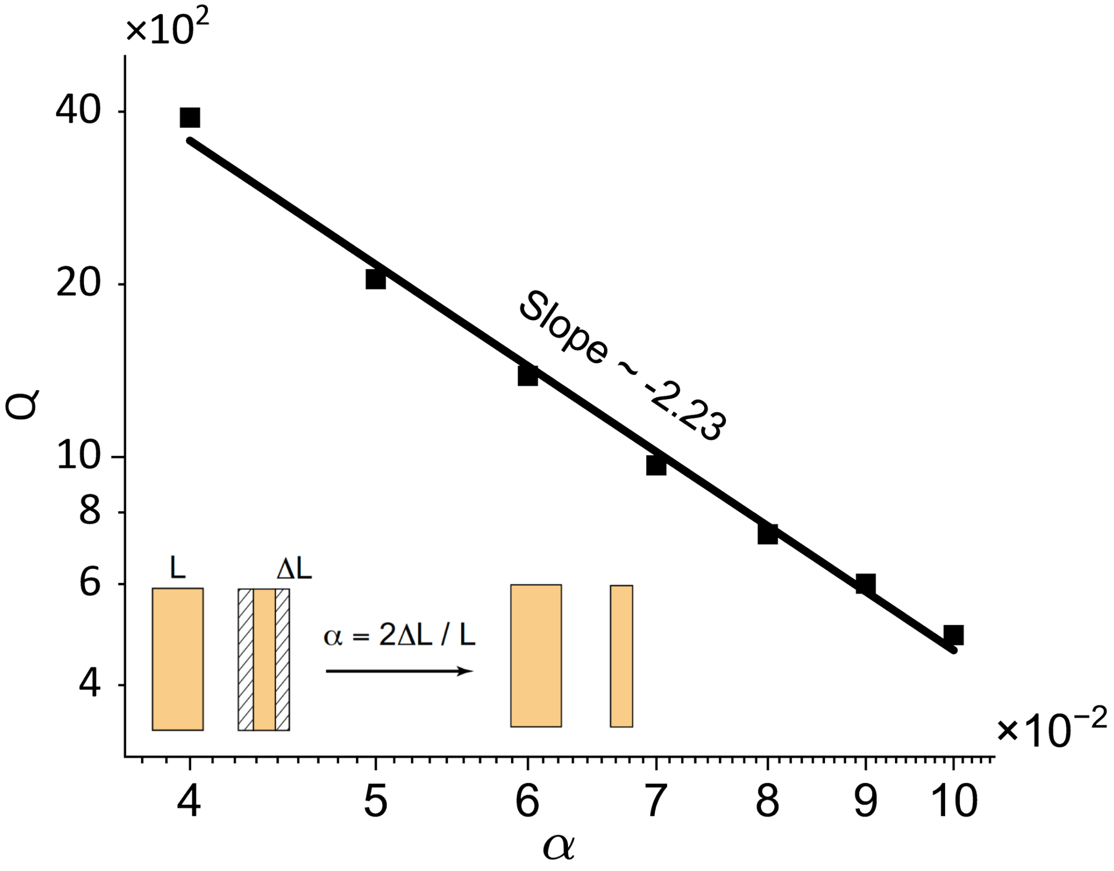

Figure 1: Radiative Q-factor dependency on asymmetry parameter α. The inset demonstrates the connection of asymmetry parameter to a width L of a nano-bar.

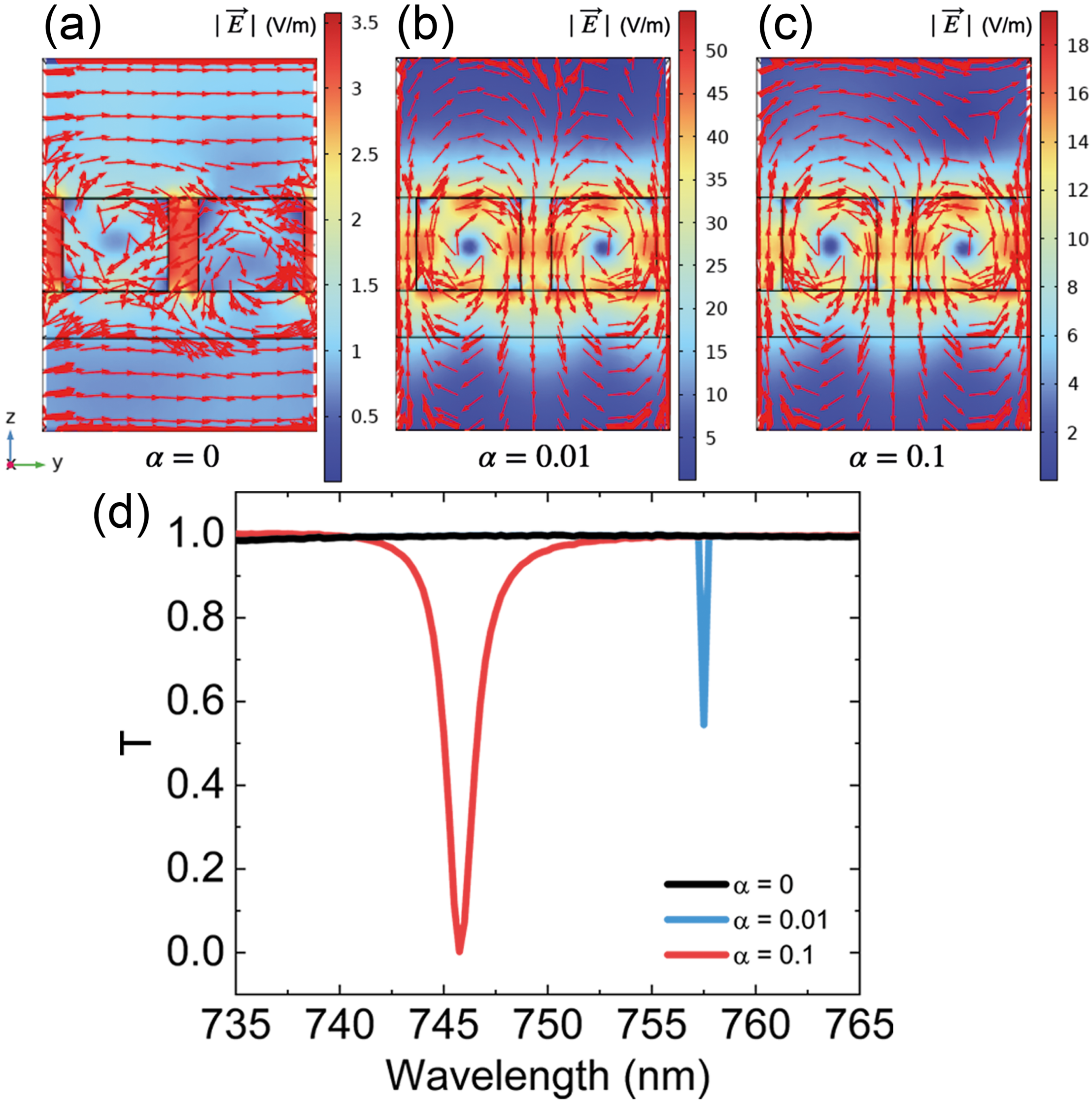

Figure 2: Calculated evolution of the near-field distribution and transmission spectrum of the q-BIC metasurface with varying asymmetry parameter $\alpha = 0$, 0.01, and 0.1: (a-c) Near-field profiles of the metasurface unit cell in the YZ-plane, that intersects the nanobars at their center, for excitation at the q-BIC resonance wavelength with a y-polarized plane wave under normal incidence. The modulus of the electric field is color coded, a snapshot of the electric field vectors is shown as red arrows. (d) Transmission spectrum evolution with asymmetry parameter values $\alpha = 0$, 0.01, and 0.1.

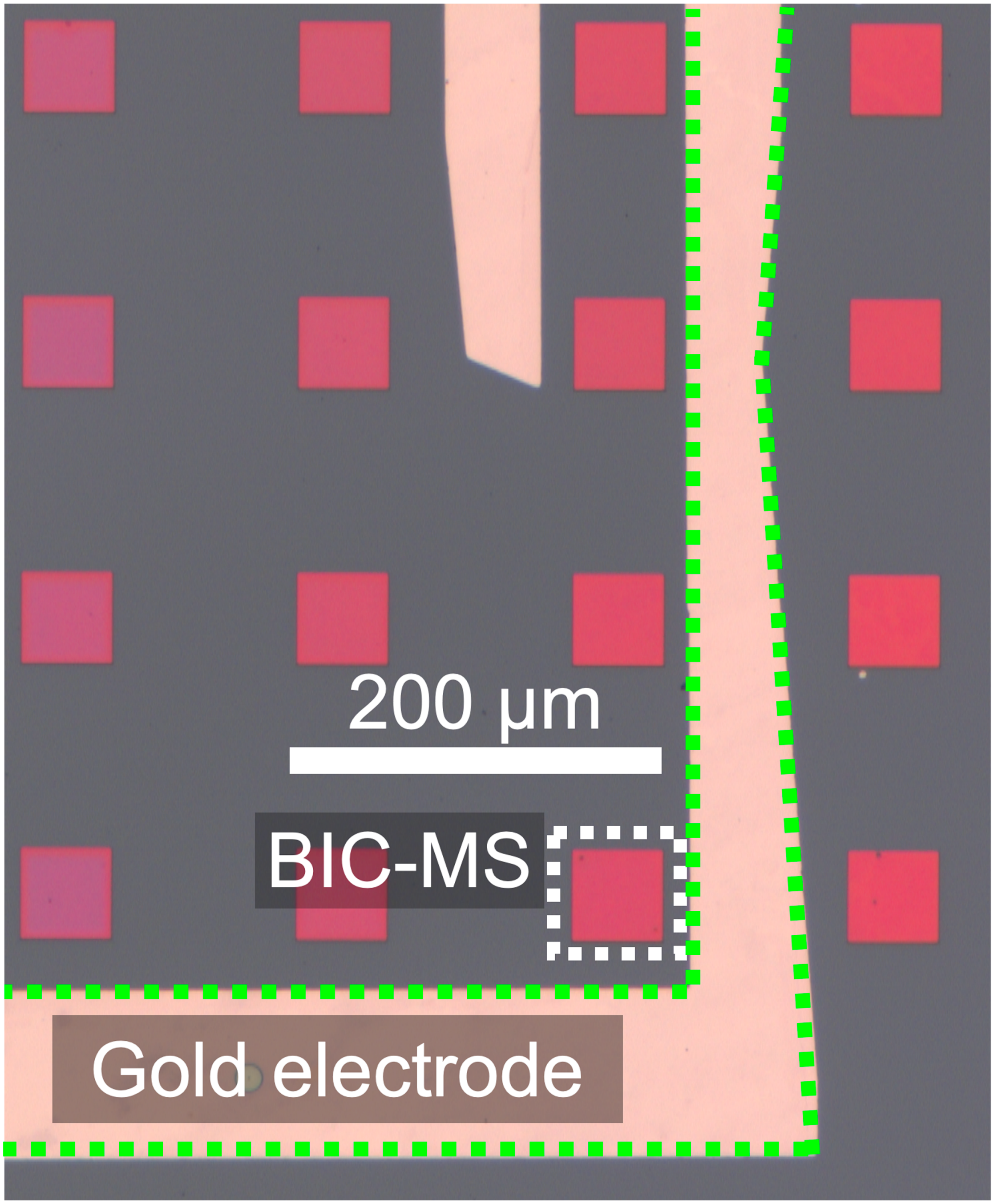

Figure 3: Bright-field microscopy image of the sample before the dry transfer step. The metasurface of interest is outlined by the white contour. The upper gold contact used in experiment is outlined by the green contour.

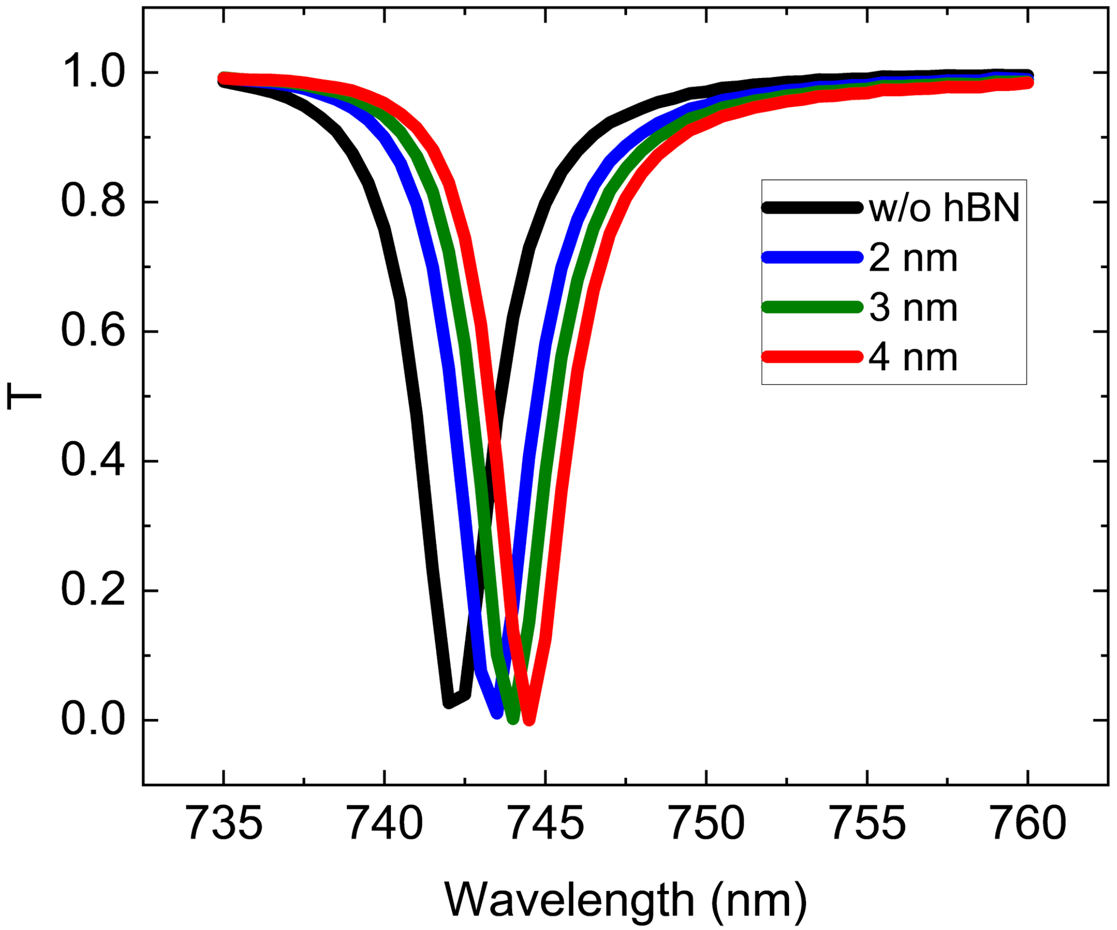

Figure 4: Simulated transmittance spectra of the bare resonant metasurface for the cases without protective hBN layer (w/o hBN), as well as for hBN layer thickness of 2 nm (blue), 3 nm (green), and 4 nm (red). The expected red shift due to the additional dielectric influence of the hBN layer for the latter case is $\simeq$2 nm.

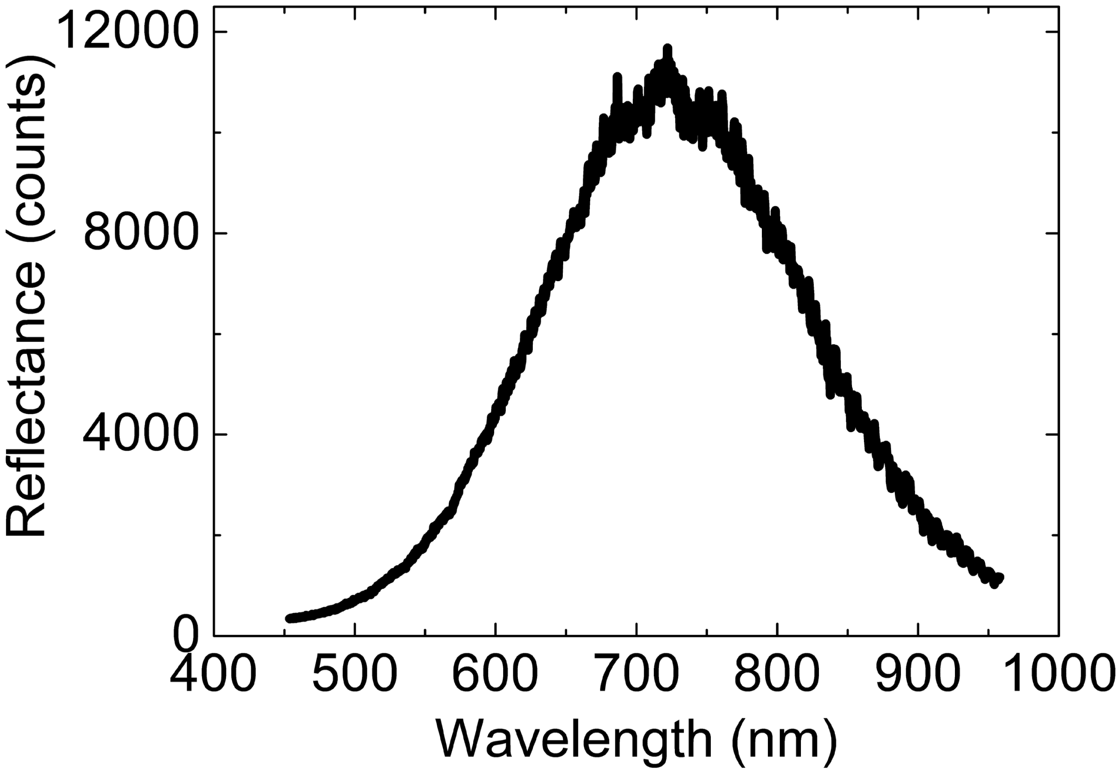

Figure 5: Measured unreferenced reflectance spectrum for the bare substrate incorporating the contribution of the white-light source, the ground electrode and fused silica substrate used as a reference signal for reflectance measurements.

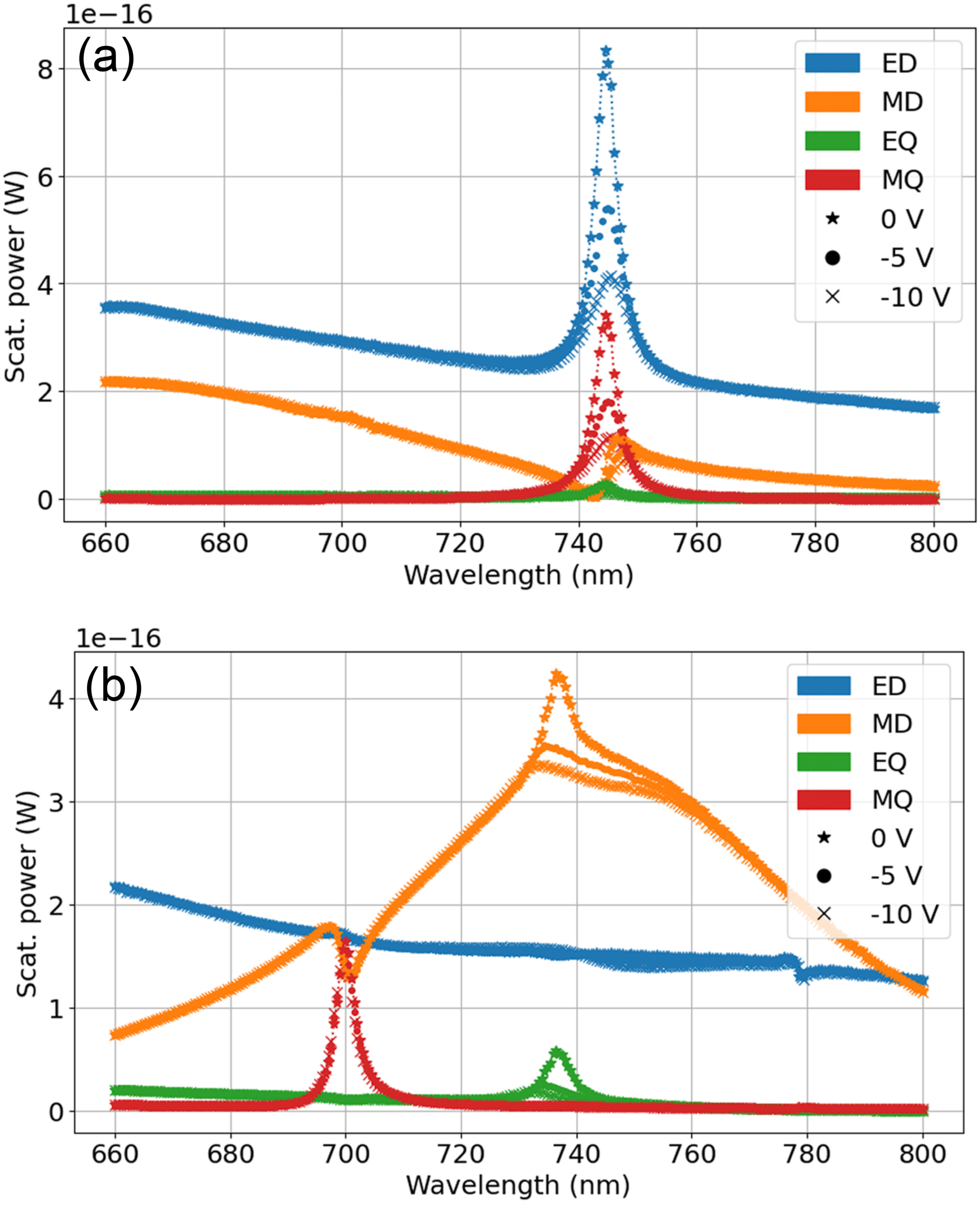

Figure 6: Calculated exact multipolar decomposition[1] for the unit cell of the hybrid metasurface. Scattering power dependencies for electric (ED), magnetic (MD) dipolar and electric (EQ), magnetic (MQ) quadrupolar contributions at the applied gate voltage values 0 V (stars), -5 V (circles), and -10 V (crosses) for (a) q-BIC and (b) off-resonant excitation types. The multipolar decomposition is conducted at the geometrical center of the unit cell of the hybrid metasurface with periodic boundary conditions.

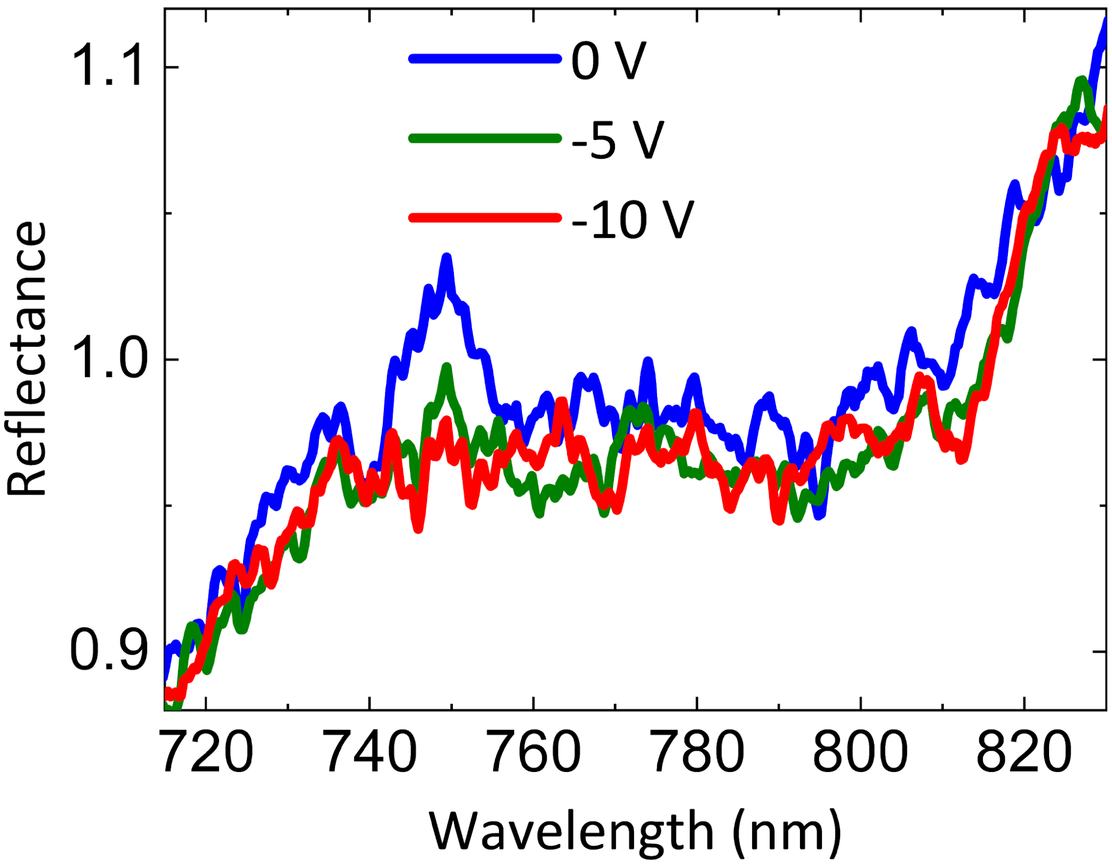

Figure 7: Experimental reflectance spectra for different external voltage values for the hBN-WSe_2 monolayer heterostructure on fused silica substrate referenced to the signal from the bare substrate.

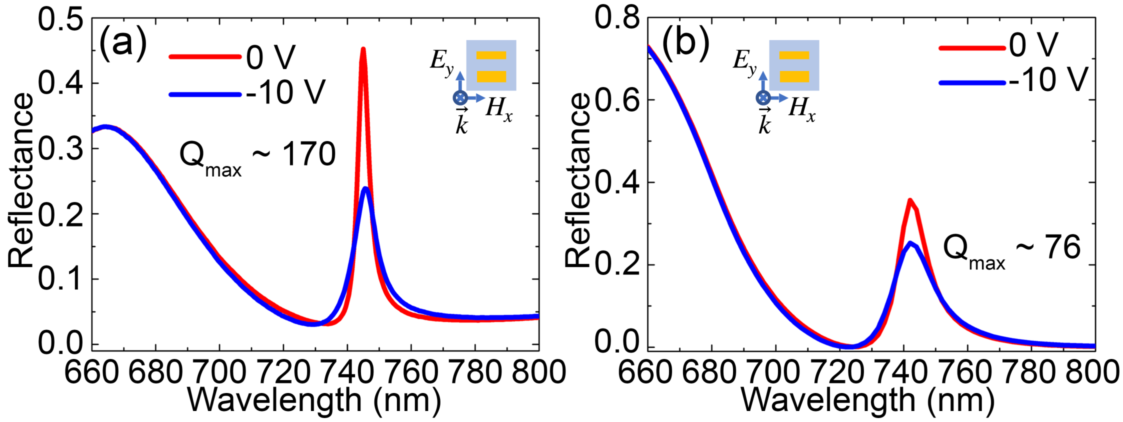

Figure 8: Simulated reflectance states at two gate voltage values (0 V and -10 V) for two different cases: (a) the ideal metasurface design with a Q-factor of 170 and (b) the metasurface design more closely reproducing the fabricated sample's Q-factor of 76. The former demonstrates the possibility of achieving an enhanced modulation depth at the level of ≈50 %, while the latter demonstrates the modulation depth limitation at 30 %.

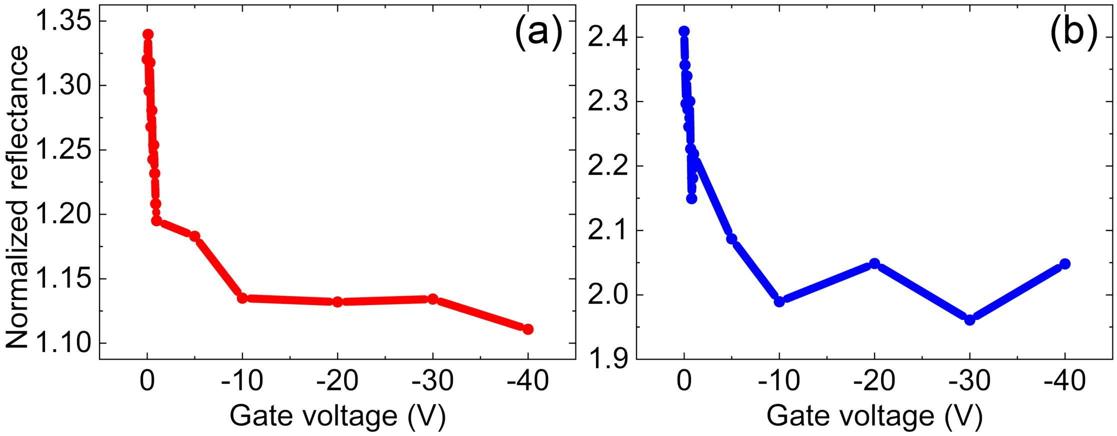

Figure 9: Measured reflectance values at 742 nm for values of the external voltage beyond -10 V for (a) q-BIC mode and (b) off-resonant excitation cases. No further functional modulation of reflectance with external voltage is observed. All reflectance values are referenced to the respective reflectance values of the bare substrate measured under the same conditions.
